# Eliciting Implicit Awareness in Alzheimer’s Disease and Mild Cognitive Impairment: A Task-Based Functional MRI Study

**DOI:** 10.3389/fnagi.2022.816648

**Published:** 2022-04-12

**Authors:** Manuela Tondelli, Francesca Benuzzi, Daniela Ballotta, Maria Angela Molinari, Annalisa Chiari, Giovanna Zamboni

**Affiliations:** ^1^Dipartimento di Scienze Biomediche, Metaboliche e Neuroscienze, Università di Modena e Reggio Emilia, Modena, Italy; ^2^U.O. Neurologia, Azienda Ospedaliera Universitaria di Modena, Modena, Italy; ^3^Dipartimento di Cure Primarie, Azienda Unitá Sanitaria Locale (AUSL) Modena, Modena, Italy

**Keywords:** anosognosia, unawareness, implicit awareness, Alzheimer’s disease, dementia

## Abstract

**Background:**

Recent models of anosognosia in dementia have suggested the existence of an implicit component of self-awareness about one’s cognitive impairment that may remain preserved and continue to regulate behavioral, affective, and cognitive responses even in people who do not show an explicit awareness of their difficulties. Behavioral studies have used different strategies to demonstrate implicit awareness in patients with anosognosia, but no neuroimaging studies have yet investigated its neural bases.

**Methods:**

Patients with amnestic mild cognitive impairment and dementia due to Alzheimer’s disease underwent functional magnetic resonance imaging (fMRI) during the execution of a color-naming task in which they were presented with neutral, negative, and dementia-related words (Dementia-Related Emotional Stroop).

**Results:**

Twenty-one patients were recruited: 12 were classified as aware and 9 as unaware according to anosognosia scales (based on clinical judgment and patient-caregiver discrepancy). Behavioral results showed that aware patients took the longest time to process dementia-related words, although differences between word types were not significant, limiting interpretation of behavioral results. Imaging results showed that patients with preserved explicit awareness had a small positive differential activation of the posterior cingulate cortex (PCC) for the dementia-related words condition compared to the negative words, suggesting attribution of emotional valence to both conditions. PCC differential activation was instead negative in unaware patients, i.e., lower for dementia-related words relative to negative-words. In addition, the more negative the differential activation, the lower was the Stroop effect measuring implicit awareness.

**Conclusion:**

Posterior cingulate cortex preserved response to dementia-related stimuli may be a marker of preserved implicit self-awareness.

## Introduction

Patients with mild cognitive impairment (MCI) and dementia due to Alzheimer’s Disease (AD) may be unaware of their cognitive and behavioral symptoms. The inability to recognize or adequately appreciate the severity of deficit in cognitive or affective functioning is termed “anosognosia” or “impaired self-awareness” (Prigatano, 2010).

Early imaging studies on anosognosia have mainly looked at correlations between clinical measurements of anosognosia and imaging variables capturing brain metabolism (such as 18-F fluorodeoxyglucose positron emission tomography, FDG-PET) and brain morphology (such as volumetric MRI) (Zamboni and Wilcock, 2011; Tondelli et al., 2018). More recent studies have related measurements of anosognosia to brain functional connectivity using resting state functional magnetic resonance imaging (fMRI) (Perrotin et al., 2015; Vannini et al., 2017; Mondragon et al., 2019). In all these studies, anosognosia was assessed at an explicit level by measuring the discrepancy between the patient’s self-report on their performance on cognitive tests with their actual performance, or between the patient’s opinion and the opinion of a caregiver or clinician (Tondelli et al., 2018). The few studies that have used task-based functional magnetic resonance imaging (fMRI) to explore mechanisms underlying anosognosia in patients with cognitive impairment have also adopted functional tasks explicitly eliciting self-reflection (Ries et al., 2007; Ruby et al., 2009; Zamboni et al., 2013b).

Nevertheless, increasing evidence has shown that some patients with cognitive impairment are able to adjust their behavior to their decreased abilities despite the presence of anosognosia at an explicit level, suggesting the persistence of mechanisms of awareness on their cognitive difficulties at an implicit level in dementia, in parallel to models of implicit awareness in anosognosia for hemiplegia (Cocchini et al., 2010; Fotopoulou et al., 2010; Geurten et al., 2021). Using an emotional Stroop paradigm, Martyr et al. found that patients with dementia as well as their caregivers showed increased response times to salient words related to dementia and forgetfulness in comparison to neutral words. Importantly, this effect in dementia patients was unrelated to the degree of awareness that they demonstrated in explicit tasks (Martyr et al., 2011). Similarly, Mograbi et al. (2012b) showed that patients with AD had preserved emotional reactivity to failure, both in terms of self-report and facial expression, despite reduced explicit awareness of performance. Based on this evidence, the notion of a possible double pathway involving implicit and explicit self-awareness has been incorporated in theoretical models of anosognosia in dementia suggesting that an implicit component, that bypasses explicit awareness, may be responsible for behavioral and affective regulation even in the absence of explicit awareness (Mograbi and Morris, 2013, 2014; Saj et al., 2013).

To our knowledge, no neuroimaging study has yet explored the neural substrates of implicit anosognosia in cognitively impaired patients. The purpose of this study was to investigate the correlates of implicit awareness with an implicit fMRI task based on a modified emotional Stroop paradigm.

## Methods

### Subjects

Patients were recruited from the Cognitive Neurology Clinic of the Azienda Ospedaliero Universitaria di Modena, Italy. Clinical diagnoses of MCI due to AD and dementia due to AD were made according to published criteria (Albert et al., 2011; McKhann et al., 2011). The degree of cognitive impairment was assessed by the Mini-Mental State Examination (MMSE, Folstein et al., 1975) and only patients with MMSE ≥ 22 were recruited. Handedness was assessed by means of the Edinburgh Inventory (Oldfield, 1971). Exclusion criteria also included the Hachinski score ≥ 4, prior, current, or past history of other neurological diseases, neurosurgery, or major psychiatric disorders (including depression), and the presence of behavioral disturbances other than anosognosia. The study was approved by the local ethics committee, and written informed consent was obtained from participants prior to the experiment, according to the Declaration of Helsinki.

### Measurement of Anosognosia

The presence of anosognosia or lack of overt awareness was assessed by means of two methods: (I) clinical judgment evaluated by Clinical Insight Rating Scale (CIRS, Ott et al., 1996), which defines 4 domains of a patient’s awareness (reason for the visit, cognitive deficits, functional deficits, and perception of the progression of deficits) rated by the examiner based on a separate interview with the patient and the caregiver on a scale from 0 (full insight) to 2 (no insight), and summed to obtain a total score between 0 and 8; (II) discrepancy score evaluated by Anosognosia Questionnaire Dementia (AQ-D, Migliorelli et al., 1995). This consists of 30 questions divided in the cognitive and behavioral section; the same questions are administered to patients (form A) and to their caregivers (form B) who are blind to the patient’s answers and the total AQ-D score is given by the difference between form B—form A. According to previous reports (Leicht et al., 2010; Tondelli et al., 2018), we classified patients with score ≥ 2 at CIRS and score ≥ 14 at AQ-D as having anosognosia, i.e., with no overt awareness of their cognitive deficits.

### Measurement of Implicit Awareness and fMRI Paradigm

**FIGURE 1:**
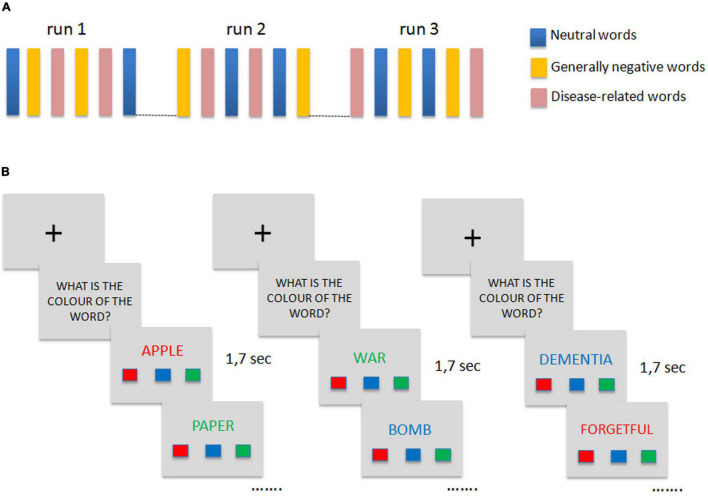
Emotional Stroop fMRI paradigm. **(A)** Graphical representation of the block-design protocol paradigm. **(B)** Examples of stimuli used in the experiment for each group of words (from left to right: neutral words, negative words, dementia-related words) and order of presentation within the block.

The task used during the fMRI experiment consisted of a modified version of the Dementia-Related Emotional Stroop used by Martyr et al. (2011), which we adapted for use as fMRI paradigm in the scanner. This is a type of Emotional Stroop test in which dementia-related words are used to test if they have greater interference (therefore greater reaction time, RT) than neutral words, thus providing a measurement of implicit awareness (Martyr et al., 2011). The task that we developed consisted of 3 experimental conditions using neutral (e.g., apple, paper, car), negative (e.g., dramatic, war, hate), and dementia-related (e.g., dementia, forgetful, disabled) words, respectively. Words were selected during a preliminary study for stimuli validation that involved 40 healthy elderly subjects (20 men, 20 women, aged between 35 and 75 years) who were asked to rate 216 words on the dimension of emotional valence (positive or negative) and relation to Alzheimer’s Disease (dementia-related or not related) using a 7 point scale (from-3 to + 3). Seventy-two words were included in the final modified version of the Dementia-Related Emotional Stroop: 24 neutral words were selected from words rating between-0.5 and 0.5 in the emotional valence questionnaire, 24 negative words were selected from those rated between-2.5 and -1 in the emotional valence questionnaire, 24 dementia-related words were selected from those words rating ≥ 1.5 in the dementia-related questionnaire, and between-2.5 and -1 in the emotional valence questionnaire to match emotional negative valence across the two groups of “emotional” words. Word types were matched on the frequency of occurrence, length, and concreteness. The fMRI paradigm was based on a block design: a total of 18 blocks of 12 neutral, negative, or dementia-related words were presented across three sessions or runs (Figure 1). The order of blocks was randomized between the sessions. Each word was presented for 1.7 s and at the beginning and at the end of each session a fixation cross was presented for 10 s. Stimuli words were arranged in a 512 × 384 pixel image using Adobe Photoshop (Adobe Systems Inc.) and were presented centrally on the screen in three different colors (red, blue, and green) on a gray background. Under each word, three colored rectangles arranged horizontally and representing red, blue, and green buttons from left to right were visible. Figure 1 graphically summarizes the experimental paradigm. Patients were instructed to press the button corresponding to the color of the word by means of an MRI-compatible button-box (Current Design Inc.); they were also instructed to press the button as fast as possible but also as correctly as possible. Accuracy and reaction time data were collected during the scanning sessions by means of a custom-made software developed in Visual Basic 6^1^. The same software was used to present stimuli. Demographical, clinical, and behavioral data were analyzed using the Stata11 software^2^ and parametric or non-parametric statistic was applied as appropriate.

### Image Acquisition and Analysis

Data were acquired with a 3T Philips Intera System scanner. Gradient echo-planar imaging T2* -weighted images were acquired (TR 2,000 ms; FOV 230 mm; 128 × 128 matrix, voxel size = 3 mm^3^). A total of 137 volumes were acquired for each session. In addition, a high resolution T1-weighted anatomical image of each subject was acquired to allow anatomical localization. The volume consisted of 170 sagittal slices (*TR* = 9.9 ms; *TE* = 4.6 ms; in plane matrix = 256 × 256; voxel size = 1 mm × 1 mm × 1 mm).

Voxel-based morphometry (VBM) was performed using T1-weighted anatomical images to determine if there were gray matter (GM) volume differences between aware and unaware patients that might account for any observed fMRI differences. VBM was performed using VBM8^3^ a toolbox of SPM8^4^. Briefly, the individual structural images were segmented into gray matter, white matter, and cerebrospinal fluid, spatially normalized to the MNI space using the DARTEL approach (Ashburner, 2007), with intensity modulation by the amount of contraction to obtain the local GM corrected for individual brain size, and spatially smoothed using a 12-mm FWHM Gaussian kernel. An independent *t*-test comparison was performed between aware and unaware patients and statistical significance was evaluated at *p* < 0.05 corrected for multiple comparisons using family-wise error correction.

A functional MRI analysis was performed using Matlab and SPM8 software (Wellcome Department of Imaging Neuroscience, London, United Kingdom). The following preprocessing steps were used: realignment to the first volume acquired, normalization to the standard SPM template, and smoothing with a 6 mm full width maximum isotropic Gaussian kernel to improve the signal-to-noise ratio. Data analysis was performed modeling three different conditions: neutral words, generally negative words, and dementia-related words. Condition effects were estimated according to the general linear model and region-specific effects were compared using several linear contrasts. Contrast images for each condition were entered into a second-level random effect analysis model and group effect (aware and unaware) was assessed by means of different two-sample *t*-test. Age, MMSE score, and disease duration were entered in the second level model as a covariance of no effect to prevent possible bias in results analyses due to disease severity or duration. A double statistical threshold (voxel-wise *p* < 0.001 and spatial extent = 47) was adopted to achieve a combined significance, corrected for multiple comparisons, of α < 0.05, as computed by 3dClustSim AFNI routine, using the “-acf” option (see details of procedure at^5^ and in Forman et al., 1995). Mean beta values were extracted from the region of interest revealed by the main analysis (disease-related vs. negative words in aware vs. unaware patients) and were plotted based on awareness classification. Mean beta values were also used to perform a correlation analysis with differential reaction time scores between disease-related vs. negative words; in this analysis, MMSE and age were entered as non-interest variable. In addition, a separate correlation analysis in all patients (irrespective of their clinical diagnosis and awareness classification) was performed between anosognosia scores measured with CIRS and functional brain response for dementia-related words (relative to negative and neutral words); age, MMSE score, and disease duration were entered in the model as covariates of no effect and a statistical threshold of uncorrected *p* < 0.001 was accepted for this follow-up analysis.

## Results

Twenty-one elderly participants took part in the fMRI study. Among them, 12 (3 AD and 9 MCI) were overtly aware of their cognitive deficits whereas 9 patients (5 AD and 4 MCI) presented anosognosia. The two groups of aware and unaware patients only differed on AQ-D and CIRS scores; no statistically significant difference was detected in the global measure of cognitive impairment and demographical features (Table 1).

**TABLE 1:** Demographic and clinical characteristics of participant.

	Aware	Unaware	Groups
	(*n* = 12)	(*n* = 9)	comparison
**Demographical and clinical characteristics**			
Gender F:M	6:6	7:2	*p* = 0.7
Age (years)	72.4 (± 6.3)	72.3 (± 7.4)	*p* = 0.9
Years of education	6.5 (3–15)	5 (4–8)	*p* = 0.1
Disease duration (years)	4.5 (3–10)	4 (2–7)	*p* = 0.5
MMSE	26.4 (± 1.2)	25 (± 3.7)	*p* = 0.08
AQ-D	7.2 (± 5.7)	23.7 (± 3.6)	***p* < 0.0001**
CIRS	0.9 (± 0.9)	6 (± 1.06)	***p* < 0.0001**

*Reported values are means with standard deviation values in parenthesis for age, MMSE, AQ-D, CIRS; reported values are median with range in parenthesis for years of education and disease duration. Comparisons between aware and unaware groups were performed with Mann–Whitney or independent t-test, as appropriate, for continuous variables and chi-square tests for dichotomous variables; a p-value < 0.008 was considered statistically significant after Bonferroni correction for multiple comparison (shown in bold).*

Reaction times (RTs) were collected for 16 subjects (9 aware and 7 with anosognosia), as the recording system failed in five subjects, limiting the possibility of subsequent classification of the subjects in implicitly aware vs. implicitly not aware on the basis of task performance. Subjects took the longest to respond to dementia-related words (mean 913 ± 205 ms), which was longer than the time they took to respond to negative (mean 900 ± 191 ms) and neutral (mean 894 ± 163 ms) words, although differences were not statistically significant (*p* = 0.466). A 2*3 mixed between-within subjects analysis of variance conducted to assess the impact of the type of words (neutral, negative, dementia-related) on subjects’ RT with and without anosognosia neither showed significant main effects of word type (*p* = 0.53) or group (*p* = 0.56), nor a significant interaction between them (*p* = 0.502). No differences in the mean number of errors for the three types of words and across the two groups of subjects were detected either.

Comparisons of structural MRI data between aware and unaware patients performed with VBM did not show any GM volume difference in the two groups, indicating the absence of potential effects that groups-specific structural differences may have had on the fMRI results.

**FIGURE 2:**
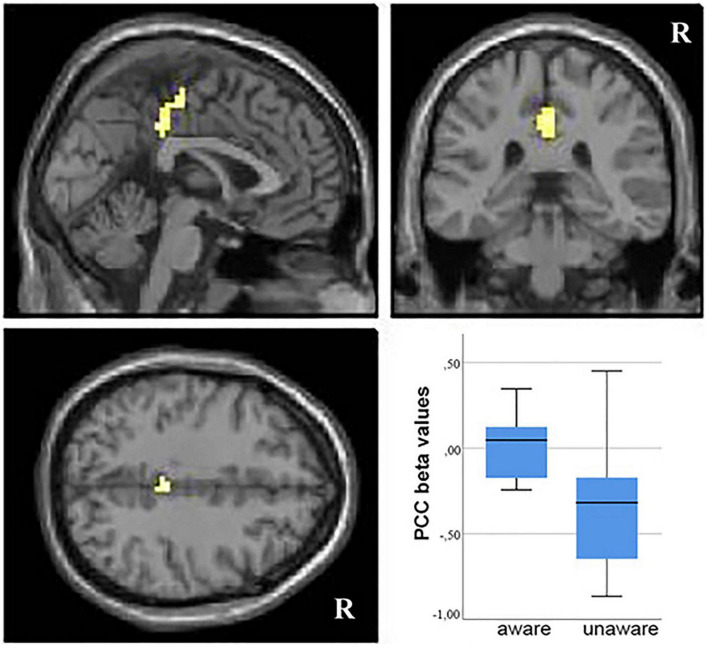
Group analysis fMRI results. Areas of increased differential signal in response to dementia-related vs. negative words in the comparison between aware vs. unaware patients (voxel-wise *p* < 0.001 and cluster size ≥ 47 voxels, as determined by 3dClustSim AFNI routine). R, right. Resulting clusters are superimposed on the MNI template implemented in SPM8. On the right, box plot of mean beta values extracted from the posterior cingulate cortex (PCC) region of interest in aware and unaware patients.

In functional MRI analyses, significant results only emerged from the comparison between groups classified on explicit awareness. Analysis of functional MRI data showed that aware patients had greater differential activation for dementia-related vs. negative words in the posterior cingulate cortex (PCC) relative to unaware patients (BA 23 and 31, MNI coordinates of peak voxel: 0, –30, 44, *Z* value = 3.82, cluster size = 55, Figure 2). Patients with preserved explicit awareness had a small positive differential activation for dementia-related vs. negative words in the resulting PCC region (mean Beta value = 0.02, *SD* = 0.19, range –0.24 to 0.35), whereas unaware patients had negative differential activation in the same region (mean beta = –0.32, *SD* = 0.38, range –0.87 to 0.45).

The same region also emerged when contrasting dementia-related words vs. neutral words in the comparison of aware relative to unaware patients. No difference in functional activity was detected in the reverse condition. When comparing negative to neutral words, no difference in neural activity was demonstrated between the two groups of patients.

The correlation analysis between mean beta values extracted from the PCC and differential reaction times between disease-related vs. negative words showed a positive correlation (*r* = 0,516; *p* = 0.054), suggesting that higher differential RT (meaning longer disease-related RT) were correlated to higher PCC activation.

**FIGURE 3:**
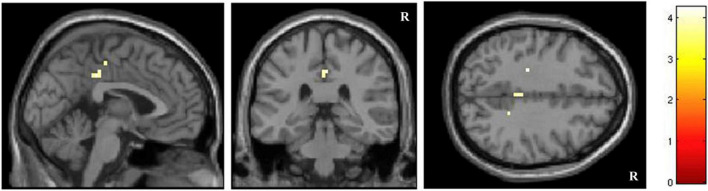
Correlation voxel-wise analysis between anosognosia scores measured with CIRS and functional brain response for dementia-related words (*p* < 0.001 uncorrected).

A correlational voxel-wise analysis across the whole brain showed that CIRS scores (high scores indicate greater severity of anosognosia) were significantly negatively correlated with brain response for dementia-related words (relative to negative words) in the PCC (MNI coordinates peak voxel: 0,–33, 37, Figure 3). This confirmed that the highest the differential activation of this region for dementia-related words, the highest the level of awareness of patients. No significant correlations emerged from the correlational analysis on disease-related relative to neutral words.

## Discussion

In this study, we investigated the neural responses involved in implicit awareness of cognitive impairment in patients with Alzheimer’s Disease by using dementia-related words in a color-naming task (Dementia-Related Emotional Stroop). We assumed that in subjects with preserved implicit awareness dementia-related words would be more emotionally salient therefore would have greater interference in the task than neutral words. We found that the difference in the activity of the posterior cingulate between experimental conditions (dementia-related words vs. neutral or negative words) was greater in patients with preserved awareness than in patients with anosognosia.

The emotional Stroop task is a variant of the Stroop test that measures the interfering properties of emotionally salient words in a color-naming task (Williams et al., 1996). The interference effect arises if the words themselves are of particular relevance to the responder or induce a feeling of threat, or if the word has a high emotional valence. The effect is thought to occur because the emotional salience of the words leads to a processing bias operating at an automatic pre-attentive processing level, with emotionally salient words subject to greater interference than neutral words (Mogg et al., 1989). Emotional Stroop tasks have been largely used to study attentional biases in people with borderline personality (Wingenfeld et al., 2009), and panic disorders (Dresler et al., 2012), but only one previous behavioral study used the emotional Stroop paradigm with dementia-related words in dementia patients (Martyr et al., 2011). The authors suggested that dementia-related words would be emotionally salient and therefore would give greater interference effect in patients with preserved implicit awareness. They demonstrated that dementia-related words elicited a processing bias in patients with dementia, since they were slower to respond to dementia-related that to neutral words.

The main result of our study was that implicit processing of dementia-related stimuli was associated with greater differential activation (relative to negative stimuli) of the PCC in the aware patients than those with anosognosia. More precisely, in patients with preserved explicit awareness, there was a small positive difference in the PCC activity between dementia-related stimuli and negative stimuli, whereas in patients with anosognosia this difference was negative, i.e., the activation for dementia-related stimuli was lower than activation for negative stimuli. Across all patients, such PCC differential activation correlated with the Stroop effect: the lower the PCC activation for dementia-related words, the smaller the difference in reaction time for disease-related relative to negative words. In other words, patients whose PCC activation for dementia-related words was comparable to their PCC activation for negative words showed a Stroop effect, a measure of implicit awareness.

A separate further imaging analysis still showed that in the PCC there was a significant voxel-wise correlation between differential activation for dementia-related relative to negative words and severity of anosognosia (measured by CIRS): the highest was CIRS, the more negative was the difference in the response of the PCC between dementia-related and negative words.

The role of PCC in the processing of emotional words is well known. Several task-based fMRI studies conducted in healthy subjects have consistently shown that the PCC is activated by stimuli with an emotional or threatening valence (Maddock et al., 2003a,b). We found that patients who activated the PCC also for dementia-related words (and not only for negative words) were those who were either explicitly aware or had a longer reaction time in response to dementia-related words (Stroop effect). Our results suggest that these patients were able to attribute an emotional or threatening valence to dementia-related words, i.e., they had preserved implicit awareness. The PCC is also a key structure of the so-called default mode network (DMN) (Raichle et al., 2001), one of the most relevant large-scale resting-state networks that can be identified with functional MRI acquired at rest, which are considered blueprints of the functional organization of the healthy (Fox and Raichle, 2007) and diseased (Zamboni et al., 2013a; Rolinski et al., 2015) brain. The DMN has been associated with self-referential processing and introspection (D’Argembeau et al., 2005; Buckner et al., 2008), as opposed to externally oriented cognitive tasks. Several studies have shown that DMN activity is also reduced in patients with AD relative to healthy controls (Greicius et al., 2004; Zamboni et al., 2013c). The hypothesis that the DMN may be the RSN whose dysfunction is associated with anosognosia in AD has been supported by recent resting-state fMRI studies (Perrotin et al., 2015; Vannini et al., 2017; Antoine et al., 2019). In particular, these studies showed an association between anosognosia and decreased functional connectivity between the PCC and the hippocampus (Perrotin et al., 2015; Vannini et al., 2017), which are both vulnerable to early AD neuropathological process (Tondelli et al., 2012). Older task-based fMRI studies conducted in healthy subjects have constantly demonstrated the activation of PCC in relation to self-appraisal (Johnson et al., 2002) and autobiographical memory (Fink et al., 1996; D’Argembeau et al., 2008). Interestingly, in the present study, we did not find significant involvement of the medial prefrontal regions during the execution of our implicit awareness task, whereas these more anterior regions have been frequently found in association with the PCC in these previous task-based fMRI studies using self-appraisal tasks (Ries et al., 2007). In particular, another task-based fMRI study among the few conducted in patients with MCI and AD showed an association between appraisal of one’s own physical, behavioral, and cognitive traits (self-appraisal) and functional activation of the medial prefrontal cortex, which was inversely correlated with measures of explicit anosognosia (Zamboni et al., 2013b). Compared to these previous task-based fMRI studies which purposefully investigated explicit domains of self-awareness by asking patients to give overt judgments on their traits, our paradigm was aimed at measuring awareness at an implicit level (Clare et al., 2011; Mograbi et al., 2012a). Thus, the PPC might represent a key structure for self-referential processing even in the absence of an explicit act of reflection about oneself. No previous study has investigated the functional correlates of implicit awareness in cognitively impaired patients, but it is plausible that in the absence of an explicit reflection about the self, the involvement of more posterior regions of the cortical midline system may emerge, whereas higher-order explicit processing may rely on more anterior regions such as the medial prefrontal cortex. Thus, in line with models of anosognosia, the PCC may possibly be a core structure for the implicit awareness pathway and serve as the sentinel node within a network involving lower/implicit and higher/explicit level mechanisms (Mograbi and Morris, 2013).

The major limitation of our study is that we did not find significant differences between experimental conditions and patient groups at the behavioral level, possibly because of the small sample size, thus limiting the interpretability of the task-based fMRI results. Nevertheless, it is not uncommon to see fMRI data in which conditions of interest elicit significant activations when contrasts are applied, even if in absence of behavioral differences between conditions. The positive correlation between PCC activation and reaction times for disease-related words (a measure of the Stroop effect), as well as the overlap of the imaging results obtained from comparisons of groups with those obtained from the correlational analysis with measures of anosognosia reconciliate the correspondence between imaging and behavior. They add confidence that our task effectively captured implicit awareness. Future studies conducted in larger numbers of patients are, nonetheless, needed to better investigate mechanisms of implicit awareness in patients with anosognosia, stratified on the basis of measures of implicit awareness. Another limitation is the heterogeneity of patients included, both AD and MCI; however, aware and anosognosic patients did not show significant differences in cognitive measures and, more importantly, VBM analysis did not show any difference in gray matter volume between the two groups, confirming that our results were not driven by structural brain difference or disease severity.

In conclusion, by using task-based fMRI with an implicit awareness paradigm in cognitively impaired patients for the first time, the present study confirmed the involvement of the PCC in mechanisms of self-awareness. Our results suggest that PCC-preserved response to dementia-related stimuli may be a marker of preserved implicit self-awareness.

## Data Availability Statement

The raw data supporting the conclusions of this article will be made available by the authors, upon request.

## Ethics Statement

The studies involving human participants were reviewed and approved by the Comitato Etico Provinciale di Modena, code 252.09. The patients/participants provided their written informed consent to participate in this study.

## Author Contributions

MT and GZ were responsible for the conceptualization, data collection, formal analysis, investigation, methodology, and writing the original draft along with FB, DB, MM, and AC who were also responsible for editing the draft. All authors contributed to the article and approved the submitted version.

## Conflict of Interest

The authors declare that the research was conducted in the absence of any commercial or financial relationships that could be construed as a potential conflict of interest.

## Publisher’s Note

All claims expressed in this article are solely those of the authors and do not necessarily represent those of their affiliated organizations, or those of the publisher, the editors and the reviewers. Any product that may be evaluated in this article, or claim that may be made by its manufacturer, is not guaranteed or endorsed by the publisher.

## Footnotes

1
http://web.tiscali.it/MarcoSerafini/stimoli_video/

2
http://www.stata.com

3
http://dbm.neuro.uni-jena.de/vbm/

4
http://www.fil.ion.ucl.ac.uk/spm/

5
https://afni.nimh.nih.gov/pub/dist/doc/program_help/3dClustSim.html
